# Avian circadian clock genes: ontogeny and role for adaptive programming in avian embryos

**DOI:** 10.3389/fphys.2025.1586580

**Published:** 2025-06-23

**Authors:** Caleb J. Wellard, Meltem Weger, Frédéric Gachon, Katherine L. Buchanan

**Affiliations:** ^1^ School of Life and Environmental Sciences, Deakin University, Geelong, VIC, Australia; ^2^ Institute for Molecular Bioscience, The University of Queensland, St Lucia, QLD, Australia; ^3^ Department of Biomedicine, Aarhus University, Aarhus, Denmark

**Keywords:** clock gene, circadian, avian ontogeny, hatching, mitochondria, melatonin, ALAN

## Abstract

Circadian clocks are ubiquitous across almost all organisms, from cyanobacteria to humans, due to a highly conserved mechanism involving a network of negative feedback loops. This molecular oscillator underpins rhythmic oscillations in physiology and behaviour at the organismal level. In vertebrates, both cellular processes and the sensory detection mechanisms underlying rhythmic physiology are relatively well understood. But how these processes develop to optimise tissue-specific rhythmic gene expression is much less understood. Birds possess an intricate, multi-oscillatory core circadian system that governs the biological rhythms of all other tissues. Avian studies document rhythmic expression of genes and hormone production prior to hatching, and yet the consequences of the onset of this process and the interactions with embryonic development have rarely been considered. In this review, we summarise the existing literature on clock gene ontogeny in birds and suggest how rhythmic expression of these genes may develop. Then, by also drawing upon evidence from non-mammalian oviparous taxa, we hypothesise how the development of rhythmic clock gene expression may interact with avian developmental processes and events. Specifically, we highlight how rhythmic clock gene expression may adaptively benefit embryos by phasing rhythms in metabolic and neuro-endocrine systems and we suggest that rhythmic gene expression may play a role in coordinating the physiological systems and behavioural outputs required to initiate hatching. Lastly, we highlight the critical avenues of research that will enhance our understanding of the role of clock genes in avian ontogeny and their ecological relevance, particularly in understanding the impacts of anthropogenic light pollution on developing avian clocks.

## Introduction

### The adaptive significance of keeping the time

Almost all organisms use the predictable cycles of light and dark caused by the rotation of Earth around its axis to time changes in behaviour and physiology according to a circadian (*circa*-around, *diem*-day) clock. To accomplish this, organisms utilise an internal cellular clock which likely first evolved around 2-3 billion years ago in cyanobacteria (Dvornyk et al., 2003). This endogenous mechanism produced rhythmicity in gene expression, which in turn drove diel variation in biological systems. The resulting rhythmic outputs have allowed organisms to detect and exploit predictable but varying opportunities for foraging and social interactions, including vulnerability to predation (Yerushalmi and Green, 2009). The core mechanistic processes were therefore broadly conserved throughout evolutionary history (Jabbur and Johnson, 2022; Helm et al., 2024). This circadian clock free-runs to a period of about 24 h, but is ‘entrained’ or re-set by external *zeitgebers* (“time giver” in German), the most potent of which is sunlight (Roenneberg et al., 2003). Such internal clocks allow two interacting benefits: they allow optimisation of behavioural patterns to changes in environmental light, whilst also programming internal physiological performance to guide optimal performance (Helm et al., 2024). Over millennia, these dual benefits at the organismal level have led to the conservation of the mechanisms driving cellular clocks across taxonomically broad groups (Jabbur and Johnson, 2022; Panda et al., 2002). During the day, diurnal animals feed, move, seek mating opportunities, and actively avoid predation and competitors, whilst at night they rest or sleep. This behavioural variation across a 24 h period is driven by diel variation in underlying physiological processes, integrated across multiple tissues, which drives organismal variation in metabolic demands, digestion, nutrient sensing, as well as motility, behaviour, and rest.

### The ontogeny of timekeeping: what is the advantage of developing a ticking clock?

In contrast to extensive studies in adult organisms, much less is known about the development of the cellular clock. Specifically, little is known about the key environmental drivers and the consequent impacts that development of rhythmic gene expression may have in directing organismal ontogeny, particularly within vertebrate groups (Vallone et al., 2007). The role of circadian genes in controlling developmental processes in mammals is difficult to disentangle due to the intrinsic interactions between maternal and offspring physiology. While several studies show that the rhythmicity in clock gene expression appears only after birth in rodents (Greiner et al., 2022; Kováčiková et al., 2006; Sládek et al., 2004), the differentiation program being inhibited by the expression of circadian clock genes (Umemura et al., 2022; Yagita et al., 2010), genetic or environmental disruption of the maternal circadian clock can alter the development (Hoshino et al., 2006; Smarr et al., 2017), physiology (Chaves et al., 2019; Cui et al., 2024; Ogo et al., 2021; Yao et al., 2025) and behaviour (Hoshino et al., 2006; Smarr et al., 2017) of offspring. In that context, oviparous species offer unique opportunities for testing the role of external environmental cues for controlling the development of the internal clock at the organismal level.

Phenotypic plasticity allows organisms to use external cues to guide, time, and stimulate their development to ensure that adult phenotypes are best adapted to the environment in which they live and reproduce (Westeberhard, 1989; Stearns, 1989). Which environmental cues they use to guide development has implications for population level processes and has been an active area of research in recent years to help understand the nature of adaptive development (Buchanan et al., 2022; Smallegange, 2022). Whilst we know that fundamental developmental processes are not halted by an absence of external environmental circadian rhythms (Vallone et al., 2007), this does not mean that rhythmic environmental cues do not subtly alter developmental trajectories for embryos by altering the onset, coordination, or amplitude of rhythmic gene expression within key tissues, which then drive oscillations in physiological processes during development.

The main aim of this review is to provide an overview of the existing literature on the ontogeny of circadian rhythms in birds, in terms of both mechanisms and outputs, and to highlight the key knowledge gaps. To provide context for avian ontogeny, we also refer to studies of other oviparous species, including fish, turtles and insects. We seek to i) highlight how rhythmic environmental cues may adaptively benefit avian embryos, specifically by driving diel variation in gene expression and subsequently in physiological systems and behavioural parameters, and ii) address the potential importance of circadian clock mechanisms at the gene, tissue, and organismal level during embryonic development for setting developmental trajectories in later life. We begin this review by discussing the circadian system of birds and the intracellular mechanisms that drive diel variation in avian biology. Then, by using the current literature, we speculate on how and when rhythmic gene expression may develop in avian embryos. We then discuss the implications: how rhythmic gene expression may drive diel variation in various embryonic systems and aid in the phasing of the transition of life stages in birds, specifically in the timing of hatching behaviours and emergence from the egg. Lastly, we conclude by summarising and explaining how the outputs of rhythmic gene expression may coordinate to adaptively drive embryonic development and discuss future directions of research to better understand the ecological consequences of developing clocks in wild birds.

## The avian circadian system

**FIGURE 1:**
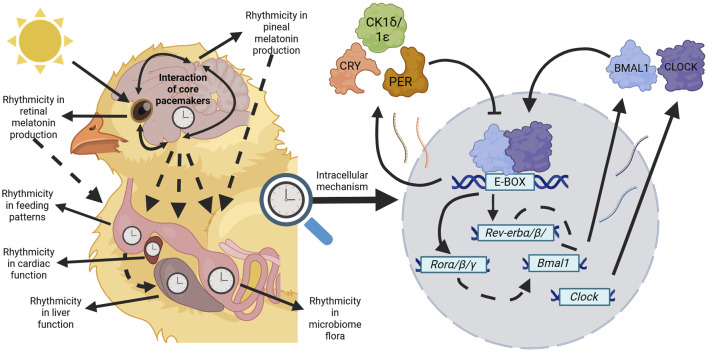
Avian circadian rhythms are orchestrated through a hierarchical, multi-oscillatory system. Core pacemakers (pineal gland, SCN, and retinae) entrain to photic cues to phase their own biological rhythms whilst also influencing other core pacemakers. Through downstream processes (dashed arrows) core pacemakers govern the biological outputs of physiological systems such as in the diversity of the microbiome or at the tissue level such as rhythmicity in cardiac parameters. Additionally, rhythms within tissues may be governed through other *zeitgebers* such as liver function entraining to organismal feeding rhythms. Regardless of the tissue type, within every cell remains the molecular mechanism influencing circadian rhythms of birds. In the core loop, *Bmal1* and *Clock* products are produced and bind to E-boxes present on gene promoters throughout the genome. Products of the negative elements (*Pers* and *Crys*) are produced and inhibit the transcriptional activity of BMAL1:CLOCK heterodimers. A stabilising loop based on the REV-ERBs and RORs nuclear receptors inhibit and stimulate, respectively, *Bmal1* and *Clock* transcription (dashed lines). This self-sustaining feedback loop lasts for approximately 24 h and maintains rhythms within all tissues of birds. Created in BioRender. Wellard, C. (2025) https://BioRender.com/d62z641.

At the organismal level, the circadian clock can be envisaged in three parts: i) the core pacemakers which generates a rhythm approximating to 24 h, ii) the peripheral clocks synchronized by the core pacemakers which coordinate timing across tissues, driving the development of coordinated rhythmic physiology and iii) the input pathways which control perception of the external cues (Menaker et al., 1978). In birds, three core pacemakers have been identified (Figure 1), these are the pineal gland (Gaston and Menaker, 1968; Zimmerman and Menaker, 1979), the suprachiasmatic nuclei (SCN) comprised of the medial and visual SCN (mSCN and vSCN respectively) (Cassone and Moore, 1987; Yoshimura et al., 2001), and the ocular retinae (Menaker, 1985; Bian et al., 2020).

### Function and diversity of the avian circadian system

The avian pineal gland intrinsically contains all elements of a circadian timing system, i.e., specialised photoreceptor cells that share properties with retinal photoreceptors for direct light sensitivity. It is also the primary site of melatonin synthesis in birds (Menaker, 1968; Falcón, 1999). In some bird species, such as pigeons (Ebihara et al., 1984) and quail (Underwood and Siopes, 1984), pineal ablation does not entirely abolish rhythms (Ebihara et al., 1984; Underwood and Siopes, 1984), as these rhythms are presumably being maintained through another core pacemaker: the SCN. The avian SCN, which is thought to sit within the hypothalamus and contain melatonin-binding sites (Lu and Cassone, 1993) (Figure 1), is likely the avian homologue to the mammalian SCN, the single central clock in mammals (Yoshimura et al., 2001). The final core pacemaker to have yet been identified in birds is the ocular retinae. Similarly to the avian pineal gland and SCN, the retina is its own independent pacemaker that functionally acts as a crucial photosensory organ for birds and intriguingly operates as a second site of melatonin synthesis for some species (Menaker, 1985). Collectively, these three core pacemakers maintain the phasing of endogenous rhythms within birds across tissues.

The avian circadian system displays remarkable interspecific variation in terms of the relative importance of pacemakers across species. For pigeon and quail species, the removal of both the pineal gland and the ocular retinae is required to induce arrhythmicity akin to pineal ablation in other species (Ebihara et al., 1984; Underwood and Siopes, 1984). This indicates that although the ocular retinae do play a role in the circadian system of birds, the relative importance of individual pacemakers is variable. Adding another layer of complexity, avian species not only show diversity between orders (e.g., *Columbiformes* and *Passeriformes*), as outlined above, but also show differences within orders. For example, pineal ablation appears to potentially have different impacts on two *Passeriformes* species. In constant darkness (DD conditions), pinealectomised house sparrows (*Passer domesticus*) lose locomotor rhythmicity whereas in pinealectomised European starlings (*Sturnus vulgaris*), locomotor rhythmicity typically persists, which the authors interpret as indicating potential differences in the importance of rhythmic melatonin synthesis on locomotor rhythms (Gwinner and Brandstatter, 2001). The mechanistic differences between species that may drive these differences, such as in the production of or the functional impact of melatonin, are not understood. These differences in the effect of melatonin are especially intriguing as the two competing, but somewhat complimentary, theories that postulate how the avian circadian system functions place melatonin as an important input/output for core oscillatory function (Cassone and Menaker, 1984; Gwinner, 1989). Interspecific differences emphasise how diverse the circadian system of birds can be, not only in structure (i.e., the relative importance of core pacemakers) but also in function (i.e., how pacemakers may interact with one another) (Gwinner and Brandstatter, 2001). Our understanding of the mechanisms underlying the avian circadian system is limited to a few domesticated/model species and yet its remarkable variability hints that there may be greater and underappreciated interspecific variation in understudied species and orders.

### The molecular mechanisms governing avian circadian rhythms

Though the avian circadian system shows diversity in its organisation, the conserved cellular mechanisms that maintain endogenous rhythms remain the same across almost all cells. In birds, the molecular clock is composed of transcriptional-translational feedback loops (Figure 1) to phase and maintain many physiological and biochemical processes, as well as subsequent behavioural outputs (Bell-Pedersen et al., 2005; Panda et al., 2002). Broadly, the components of this mechanism can be separated into positive (activator) and negative (repressor) components. The components of the positive limb (*Bmal1* and its dimerization partners *Clock* and *Npas2*) are transcribed and translated, whereby the translated products form heterodimers in the cytoplasm and enter the nucleus (Bell-Pedersen et al., 2005; Laothamatas et al., 2023; Patke et al., 2020). The presence of the positive products stimulates the transcription of the negative components (*Per* and *Cry* genes) via E-box motifs on the promoter of these genes at BMAL1-binding sites. The negative products are produced, dimerise in the cytoplasm, are modified by several kinases, including casein kinase 1δ/1ε, and re-enter the nucleus to interfere with BMAL1 heterodimers and inhibit their transcriptional activity. A secondary stabilising loop containing the genes *Rev-erbα*/*β* and *Rorα*/*β*/*γ* influences the core loop by inhibiting and stimulating *Bmal1* and *Clock* transcription, respectively (Bell-Pedersen et al., 2005; Laothamatas et al., 2023; Patke et al., 2020). This cycle continues until the positive limb is completely inhibited, whereby repressor elements are degraded through ubiquitin-proteasome pathways allowing the activator elements to recommence transcription and the cycle begins all over again. In free running conditions, meaning in the absence of environmental cues, this process runs for approximately 24 h (Bell-Pedersen et al., 2005). This mode of regulating gene expression allows for synchronous rhythmic expression of thousands of different genes in the same phase cycle, all with the aim of optimising metabolism and physiology under predictable cycles of nutrient uptake and requirement (Panda, 2016; Bass, 2024).

Functional clock genes have been identified in a handful of avian (precocial and altricial) species such as chickens (*Gallus gallus*) (Yamamoto et al., 2001; Liu et al., 2019; Bailey et al., 2003), Japanese quail (*Coturnix japonica*) (Yoshimura et al., 2000; Yasuo et al., 2003), tree sparrows (*Passer montanus*) (Renthlei et al., 2019), and house sparrows (Helfer et al., 2006; Abraham et al., 2003). The avian orthologs of the core clock genes share sequence similarities to those of mammals and yet show distinct differences (Yoshimura et al., 2000). For example, like mammals, birds possess *Per2* and *Per3* genes, however, they do not express a *Per1* ortholog (Bailey et al., 2003; Bailey et al., 2004; Yoshimura et al., 2000). Though inter-taxonomic variation has arisen in clock genes across evolutionary history, ultimately and ubiquitously, rhythmic gene expression at the cellular level forms the basis for controlling temporal patterns across tissues, in organismal level physiological and behavioural rhythms.

### Ontogeny: clock development in non-avian oviparous vertebrates

Oviparous species offer an opportunity to quantify the impact of environmental conditions on the ontogeny of clock gene expression. But we know very little about the ontogeny of expression of these genes in embryonic birds or what stimuli drive this development. However, some of the most relevant studies we can draw from to speculate how they may develop have been conducted on zebrafish (*Danio rerio*), a small fish vertebrate development model. Early investigations suggested that zebrafish embryos inherit a functional circadian clock maternally (Delaunay et al., 2000), but this has been debunked, with the current view that circadian clocks and their outputs gradually develop during embryogenesis. Circadian clock components are present early in zebrafish embryos during development, specifically as maternally inherited clock transcripts that are later replaced by zygotic transcripts on embryonic day (ED) 1 (Dekens and Whitmore, 2008). However, the earliest clock gene oscillation, observed for *per1* in whole embryo extracts, does not begin until ED2, while *clock* and *bmal1* oscillations start even later, at ED4 (Dekens and Whitmore, 2008). This gradual development and maturation of functional circadian clock oscillations is further supported by *in vivo* studies using luciferase reporter lines to monitor *per3* (Kaneko and Cahill, 2005) and circadian core clock activity (Weger et al., 2013) at the whole embryo/larvae level. While to date not much is known, it is assumed that an operational circadian clock during early embryogenesis may be critical for its own proper maturation and regulatory functions in developmental processes (Laranjeiro and Whitmore, 2014; Weger et al., 2017).

The transcriptional onset of circadian clock transcripts in the zebrafish embryo is autonomous (Dekens and Whitmore, 2008) and occurs without the need of any entrainment signals such as light-dark or temperature cycles. But circadian rhythms of clock gene expression (Dekens and Whitmore, 2008), activity (Weger et al., 2013), and outputs (Dekens et al., 2003; Gothilf et al., 1999; Hurd and Cahill, 2002; Kazimi and Cahill, 1999; Ziv and Gothilf, 2006) cannot be detected at the population level when entrainment signals are absent. This observation clearly highlights that while entrainment signals are not required for the initiation of a functional circadian oscillator (Dekens and Whitmore, 2008; Weger et al., 2013), they are necessary to reset and synchronise all oscillators between individual cells, which initially oscillate asynchronously. Ultimately, the development of rhythmic gene expression may drive developmental changes in cellular processes and cycles (Figure 3b), physiology (Figure 3c) and metabolism (Figure 3d), the outputs of which would vary depending on the *zietgebers* present across nesting strategies and conditions.

### Ontogeny: how and when do avian embryos begin entrainment?

Throughout incubation, avian embryos may be exposed to a variety of rhythmic cues to entrain their rhythms to, the strongest of which would be light. Birds possess several extraocular photoreceptors in several areas of the brain including the pineal gland (Korf, 1994), the telencephalon (Wang and Wingfield, 2011), but crucially also the deep brain in the medio-basal hypothalamus (García-Fernández et al., 2015; Meddle and Follett, 1997) expressing a variety of opsins (Pérez et al., 2019). In addition, there is intriguing evidence that embryonic chick iris striated muscle may also possess photosensitivity through photosensitive CRY proteins that are usually found in insects and which may be a property of other striated muscle development (Margiotta and Howard, 2020), although this remains to be demonstrated. In most insects, photosensitive CRY is required to synchronize the clock through the photosensitive degradation of the PER protein and the negative branch of the circadian clock (Ceriani et al., 1999; Emery et al., 1998). Intriguingly, though the function of some CRYs may vary across taxonomic groups (Stanton et al., 2022), some CRYs are conserved and shared, yet with minor changes across groups such as mammalian-like non light sensitive CRY (MCRY) across insects and vertebrates (Deppisch et al., 2023). These deep brain photoreceptors likely play an important, but yet undetermined, role in coordinating rhythmic gene expression in the developing bird. In adult birds, changes in photoperiod are detected by these deep brain photoreceptors (García-Fernández et al., 2015), which have been lost in mammals (discussed further in Gerkema et al., 2013). Therefore, whilst the physiological response to changing photoperiod is conserved across birds and mammals, both the cellular responses and the method of detection are different (García-Fernández et al., 2015). Considering these functional sensory systems are present in embryonic birds, the onset of rhythmic gene expression might occur earlier in development in birds than mammals, as documented in fish (Dekens and Whitmore, 2008; Kaneko and Cahill, 2005; Weger et al., 2013). Yet, despite clear evidence for the capacity of avian embryos to detect light changes, it remains unclear exactly when avian embryos (either precocial or altricial species) start to entrain to rhythmic light cycles.

It is worth emphasising that there is evidence that embryonic birds possess the ability of non-photic entrainment (i.e., entrainment to *zeitgebers* that are not light). Specifically, embryonic entrainment appears to occur in chicken embryos in relation to rhythmic changes in temperature (Zeman et al., 2004), a trait which also described in zebrafish (Lahiri et al., 2005; Villamizar et al., 2012). Chick embryos held in DD under a thermocycle varying by 4.5 C (16:8) over the second half of their incubation are able to entrain their melatonin production (Zeman et al., 2004). Embryos showed increased melatonin production during the low temperature phase, with pineal melatonin levels declining before the temperature rise. Furthermore, these cycles persisted in the last 2 days prior to hatching in embryos entrained to this thermocycle ED10-ED19 but then kept in constant temperature (Zeman et al., 2004). This intriguing phenomenon has received remarkably little research attention, but the biological relevance of diurnal changes in ambient temperature for ectothermic vertebrates suggests that embryos could potentially adaptively program their physiological processes to anticipate the likely environmental conditions (Mariette and Buchanan, 2016). Avian embryos may naturally experience rhythmic thermal changes when parents remove themselves from the eggs or from natural changes in the ambient temperature. Yet, to our knowledge, there are no published studies which have quantified clock gene entrainment in avian embryos in relation to a temperature cue.

### How might the ontogeny of embryonic clock gene expression affect development?

**FIGURE 2:**
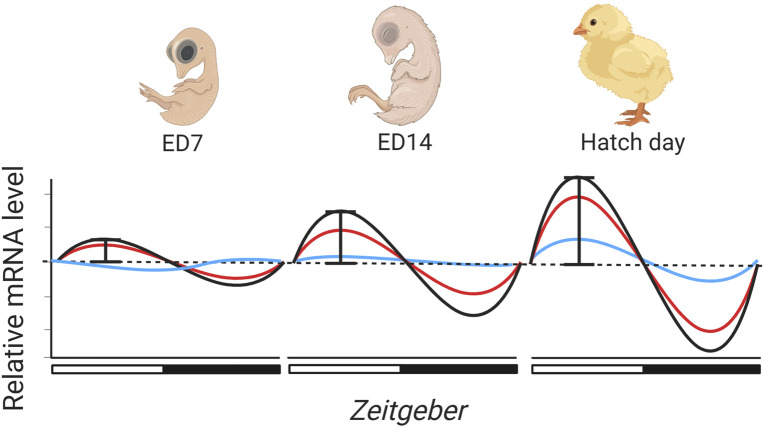
Rhythmic light cycles throughout incubation may drive the ontogeny of clock gene expression in avian embryonic development. Subsequently, the molecular clock in avian embryos may gradually develop and mature, though only a handful of studies have tested the effects of incubation conditions on rhythmic clock gene development (Table 1). From the literature, we predict that the onset of rhythmic gene expression will commence earlier in core pacemakers (red and black lines) than in peripheral organs (blue line), but also that the amplitude of expression will be greater. Light may synchronise rhythmic expression so that by hatch day clock gene expression is synchronised across cells/tissues. There is an indication that the avian clock may be autonomous, however, we speculate that rhythmic cycles of light may be important in synchronizing and entraining clock gene expression earlier than it would be in free-running conditions. Created in BioRender. Wellard, C. (2025) https://BioRender.com/v67e215.

There are a number of different plausible ways in which the ontogeny of avian clock gene expression may adaptively allow embryos to phase developmental processes and events. To understand the potential for this to occur, we need to understand the patterns in development of expression across embryonic time and space. The findings of a handful of published studies to date document the likely patterns of the onset of tissue-specific rhythmic avian clock gene expression (Table 1). These studies suggest that the onset of rhythmic gene expression and its amplitude will differ across embryonic tissues (Figure 2). Due to the importance of circadian pacemakers in circadian timekeeping, and akin to what is alluded to in zebrafish (Ziv and Gothilf, 2006), the onset of rhythmic core clock gene expression in pacemakers will occur prior to the onset of cycling in peripheral tissues. Tissue-specific differences in phasing suggest that core pacemakers drive the progression of phased gene expression across the body. This progression of rhythmic expression and phasing of cycles across tissues is already alluded to in the literature, as rhythmicity in the expression of most clock genes is not apparent in peripheral tissues in mid-late-staged chick embryos (Herichová et al., 2006; Zeman et al., 2009), although diurnal variation (Okabayashi et al., 2003) and rhythmicity (Kommedal et al., 2013; Herichová et al., 2008; Csernus et al., 2007) in clock gene expression appears in core pacemakers at the same stage. Though interestingly, although some clock genes do not rhythmically express in ED18 chick embryos (Zeman et al., 2009), recent observations suggest the presence of diurnal lipid metabolism in light-entrained ED18 embryos (Wang et al., 2024). This intriguingly indicates that arrhythmicity in some clock genes may not be entirely impairing for avian embryonic tissue functions to change across the day.

**TABLE 1:** Studies documenting clock gene ontogeny throughout avian embryonic development; studies use varying lighting conditions and not all studies reported details on light exposure. All studies were completed on chicken model.

Paper	Study system	Tissue	Developmental period	Target clock genes	Lighting conditions	Main findings
Gonçalves et al. (2012)	*In vivo*	Whole embryo (early somite stage)	Blastula and gastrula	*Clock* and *Bmal1*	0:24DD	*Clock* and *Bmal1* expression were identified throughout this period. Cyclic expression not tested.
Nagy et al. (2009)	*In vivo* and *in vitro*	Pineal gland	ED14-ED19	*Clock* and *Cry1*	0:24DD	Displayed autonomous diurnal variation in mid-late staged tissues. *In vivo*: *Clock* and *Cry1* begin oscillations in expression at ED17. *In vitro*: *Clock* and *Cry1* begin oscillations in expression at ED14.
Okabayashi et al. (2003)	*In vivo*	Pineal gland and SCN	ED15-ED21	*Per2*	12:12LD or 0:24DD	SCN: *Per2* expression was significantly higher in the middle of the light period compared to the dark period from ED16. Pineal: *Per2* expression was significantly higher in the middle of the light period compared to the dark period from ED18. No differences in *Per2* expression between time points in DD embryos across both tissues. Cyclic expression not tested.
Herichová et al. (2008)	*In vivo*	Pineal gland	ED19	*Per2 and Bmal1*	12:12LD or 0:24DD	*Per2* displayed significant changes in expression in LD but experienced dampened expression when incubated in DD after entrainment. *Bmal1* displayed weak diurnal variation in gene expression in both LD and DD and expression was similar across both incubation treatments.
Herichová et al. (2006)	*In vivo*	Heart	ED19	*Per2*	12:12LD	*Per2* expression was arrhythmic.
Zeman et al. (2009)	*In vivo*	Heart and liver	ED19	*Per2*and *Bmal1*	12:12LD	*Per2* and *Bmal1* expression were arhythmic in both tissues but *Per2* displayed diurnal variation in expression across both tissues.
Wang et al. (2024)	*In vivo*	Liver	ED18	*Clock*	12:12LD	*Clock* displayed rhythmic expression.
Kommedal et al. (2013)	*In vivo*	Pineal gland	ED19-ED21	*Clock*	12:12LD or 0:24DD (post entrainment)	*Clock* was displayed significant diurnal variation in expression under LD conditions. *Clock* continued robust diurnal variation once placed in DD.
de Lima et al. (2011)	*In vitro*	Retinae	ED8 (sampled after 6 days *in vitro*)	*Clock and Per2*	0:24DD, 12:12LD or 3 days 12:12LD + 3 days 0:24DD (post entrainment)	0:24DD: No clock genes show rhythmic expression but exhibited diurnal variation. 12:12LD: Both *Clock* and *Per2* displayed diurnal variation in expression. 12:12LD + 0:24DD: Both *Clock* and *Per2* displayed no significant differences in their expression levels across timepoints.
Nagy and Csernus (2007)	*In vitro and* *in vivo*	Pineal gland	ED17-ED18	*Clock and Cry1*	0:24	*In vivo*: Expression of *Clock* showed weak diurnal variation and *Cry1* expression displayed significant changes in expression. *In vitro*: Expression of *Clock* and *Cry1* displayed significant diurnal variation.
Csernus et al. (2007)	*In vivo*	Pineal gland	ED18-19	*Cry1*	12:12LD	*Cry1* displayed diurnal variation.

The timing of the onset of rhythmicity in clock gene expression seems likely to vary across species, with precocial species likely to show higher amplitude and more coordinated rhythms in expression levels than altricial species which hatch at a less developed stage. In zebrafish (Dekens and Whitmore, 2008) rhythmicity is autonomous, but whether rhythmic expression develops without external cues is unknown for birds. Early staged chicken blastula and gastrula display *Clock* and *Bmal1* expression, indicating that autonomous clock gene expression begins early in avian embryogenesis (Gonçalves et al., 2012). In DD conditions, *Cry1* and *Clock* expression in ED17 (*in vivo*) and ED14 (*in vitro*) pineal cells display autonomous diurnal variation (Nagy et al., 2009). Additionally, ED15-ED21 chick embryos do not display diurnal variation in *Per2* expression between two time points in the SCN and pineal, and absolute *Per2* expression levels also do not increase across developmental days in DD (Okabayashi et al., 2003). Collectively these studies show that under DD conditions, i) the molecular clock begins expression in the early stages of embryonic development, ii) the molecular clock does exhibit autonomous expression, and iii) across developmental days, clock gene expression levels appear to remain at a constant level with no increase in gene expression. It is also unknown whether cyclic light patterns influence the amplitude of rhythmic clock gene expression across avian development. This is potentially alluded to as ED15-ED21 chick embryos incubated under rhythmic patterns of light and darkness (LD conditions) experience significant and increasing diurnal variation in *Per2* expression across development (Okabayashi et al., 2003). This indicates that both absolute clock gene expression levels and cyclic amplitude may indeed gradually develop across development (Figure 2). However, to our knowledge, it is yet to be explicitly tested how cyclic light patterns influence the amplitude of clock gene expression across embryogenesis.

Most avian embryos develop in relatively dark environments, with exposure to pulses of light when incubation is broken, with few eggs naturally experiencing complete DD throughout the incubation period. Experimental studies suggest that DD might result in reduced phase synchronicity in gene expression across cells and tissues. Studies of adult birds have demonstrated that clock gene rhythmicity and phase synchrony are indeed reduced when birds experience DD conditions, though the effect is variable among clock genes (Abraham et al., 2002; Yoshimura et al., 2000). The ecological relevance of any change to the onset and amplitude of expression in developing avian embryos remains unaddressed, but one could speculate that patterns of rhythmic expression may differ between open cup and cavity/burrow nesting species where light exposure will naturally vary considerably during incubation.

The onset of rhythmic clock gene expression may coincide with tissue-specific functions at specific developmental stages. If so, factors that affect the onset or amplitude of gene expression may have impacts on developmental trajectories. For example, rhythmic melatonin synthesis seems likely only to follow rhythmicity in clock gene expression within the pineal gland and retina, whilst rhythmic melatonin synthesis may well affect the phenotype at hatching (Helm et al., 2024). Future studies are needed to assess the factors determining the onset, amplitude, and organismal coordination of rhythmic gene expression, as well as the developmental consequences for avian embryos.

### Circadian rhythms in avian embryonic endocrine systems

There are strong circadian rhythms in avian endocrine function (discussed further in Helm et al. (2024)), which regulate behavioural and physiological functions, driven by rhythmic expression of a range of genes including clock genes. In all vertebrates, the hypothalamic-pituitary-adrenal (HPA) axis shows strong diurnal rhythms, with the highest levels of circulating glucocorticoids (GC) occurring just before the start of the active period, in anticipation of the likely changes to metabolic activity (Gotlieb et al., 2018). These diurnal rhythms in endocrine function are also true in birds (Breuner et al., 1999; Rich and Romero, 2001; Schwabl et al., 2016). The impact on diurnal physiology is better studied in mammals than in birds: the loss of *Bmal1* in corticotropin releasing hormone (CRH) neurons results in the cessation of any daily rhythm of circulating cortisol in mice (Jones et al., 2021). GCs (in birds mostly corticosterone) are produced by the adrenal gland upon stimulation by both adrenocorticotropic hormone (ACTH), which is produced in the anterior pituitary (Blas, 2015). In rats, removal of the pituitary, which would abolish ACTH production, does not alter clock gene rhythmicity in the adrenal cortex (Fahrenkrug et al., 2008) suggesting that in mammals at least, the SCN plays a fundamental role in determining the circadian rhythm within adrenal function, including its activation by light signals (Ishida et al., 2005).

Whilst circadian gene expression affects the establishment of diurnal patterns of neuroendocrine activity, with resulting behavioural and physiological outputs, it is also possible that early life experiences may affect the establishment of the circadian clock. For example, Harvey-Carroll et al. show that experimental administration of corticosterone to Japanese quail (*Coturnix coturnix*) early stage embryos altered expression of *Bmal1* and *Per2* within the hypothalamus, in a time and developmental stage-specific manner (Harvey-Carroll et al., 2024). These differences were apparent after hatch and linked to changes in activity and feeding behaviour in a sex-specific manner (Harvey-Carroll et al., 2024). These interesting data suggest that early life events may dictate the development of the circadian clock rhythms which, in turn, affect development, physiology, behaviour, and potentially individual fitness.

Melatonin is a fundamental hormone for determining and maintaining diurnal patterns of activity and rest. In birds, it is released from the pineal gland and retinae in response to darkness, with levels peaking in the middle of the night (Helm et al., 2024; Jones et al., 2015; Whittow, 1999). Melatonin binding sites have been identified in the retinae, as well as a range of sites within the brain associated with visual processing, including the vSCN and the optic tectum, suggesting a role for melatonin in visual sensitivity and processing (Cassone, 2014). In some songbird species, melatonin binding is seen within the song control nuclei (Gahr and Kosar, 1996), which may be functionally significant for vocal learning (Jansen et al., 2005). It is known that melatonin coordinates both daily activities and in the longer term organises the timing of the annual cycle (Cassone, 2014; Helm et al., 2024). In contrast to the lack of any diurnal rhythm in corticosterone levels (Huffeldt et al., 2020), both thick-billed murres (Huffeldt et al., 2020) and Lapland longspurs (*Calcarius lapponicus*) (Ashley et al., 2014) breeding in polar regions under continuous light maintain rhythmic, low-amplitude melatonin production.

To date, most experimental studies testing avian embryonic endocrine rhythms have focused on quantifying melatonin production (further reviewed in Helm et al. (2024), Zeman and Herichová (2011)). Intriguingly, melatonin may play a programmatic role in early avian development (Helm et al., 2024) because in at least some precocial bird species, unlike humans which only show rhythmic melatonin production after birth (Kennaway et al., 1992), synthesis begins well before hatching (Hanuszewska et al., 2019; Lamošová et al., 1995). Rhythmic expression of arylalkylamine N-acetyltransferase (AANAT), the penultimate and rate-limiting enzyme in melatonin synthesis, is present in chicken embryos by ED16, with increasing rhythmic amplitude as hatching approaches (Zeman et al., 1999). In addition, rhythmicity in N-Acetylserotonin O-methyltransferase (*Hiomt*), a key enzyme for the final stage of melatonin production, is observed in ED19-21 chick embryos (Kommedal et al., 2013). In this second half of embryonic life, rhythmic melatonin levels become entrained to environmental light cycles and rhythms persist even in the absence of light (Zeman et al., 1999). Interestingly, *in vitro* dispersed cultured chicken pineal gland cells show rhythmic melatonin production from around ED16 (Zeman and Herichová, 2011), which dampen in dispersed cell culture after 48 h of DD (Lamošová et al., 1995). These data suggest that whilst cellular rhythms within the pineal gland cells are initially autonomous, coordination is needed between cells and across tissues over time to maintain entrainment. Whilst rhythmic melatonin production has not been documented in altricial starlings (Zeman and Gwinner, 1993) or zebra finches (Van’t Hof and Gwinner, 1996) until after hatching, it is possible that this is due only to lack of experimental focus. Indeed, hatchling starlings were found to have both pineal and plasma melatonin levels which matched the expectations given the sampling time and prehatch light environments (Gwinner et al., 1997). These data strongly suggest that light cycles have an important role in determining embryonic developmental pathways, even well before hatching in altricial bird species. There is some suggestion that combining environmental cycles of both light and temperature during chicken incubation may provide additive impact effects on rhythmic cycling of AANAT levels and therefore possibly melatonin synthesis (Yalcin et al., 2022; Zeman et al., 2004). The development of prehatch melatonin rhythms raises the question of whether early embryonic environmental cues may program physiological rhythms, which then have consequences for post-hatch developmental trajectories. For example, in adult non-mammalian vertebrates, circadian melatonin production is synchronised with diurnal temperature changes (further discussed in Zeman et al. (2004)). Specifically in birds, melatonin plays an important role in thermoregulation, enabling thermal homeostasis across days and seasons (Ruuskanen et al., 2021). Similarly, avian embryos may use rhythmic changes in the external ambient temperature to set thermoregulation expectations across the day and potentially for post-hatch temperature. Hence, changes in thermal responses, which might prepare an embryo to emerge in particular environments, may start to occur even before hatching (Mariette and Buchanan, 2016).

### Circadian rhythms in avian embryonic metabolic systems

Whole organism metabolism is the sum of chemical reactions that allow animals to break down molecules, sustain cellular physiology, generate energy, and maintain normal internal physiology. Rhythmicity and diurnal variation in adult avian metabolic rhythms (e.g., neural activity, gas exchange, catabolic and anabolic processes, etc.) provide the basis for maintaining homeostasis across the day and optimising organismal function (Cassone, 2014; Panda, 2016). As animals alternate between feeding and fasting during periods of wakefulness and rest, interactions between the molecular clock and metabolism have arisen to maintain homeostasis. Rhythmicity in metabolic rate is not just shaped through external *zeitgebers* but also strongly by rhythmicity in movement (energy expenditure, body temperature) and feeding opportunities (energy source intake), which in turn can entrain peripheral oscillators such as in muscle cells (Harfmann et al., 2015) and the liver (Stokkan et al., 2001). Lines of knock out (KO) clock gene rodents demonstrate this, as they experience a plethora of metabolic effects (Bolshette et al., 2023; Hepler and Bass, 2023; Lal et al., 2024; Schrader et al., 2024), leading to early aging and shorter life expectancy (Kondratov et al., 2006; Lee et al., 2010).

To date there has been few attempts to quantify any circadian rhythms in metabolism or nutrient uptake in avian embryos. We would however suggest that metabolic rate is unlikely to be constant, particularly at later stages of development. For example, house sparrow embryos incubated under a temperate photoperiod (16L:8D) had lower rates of CO_2_ production during the night phase than they did during the light phase (Cooper et al., 2011). This was not just observed on one developmental day, but rather across 10 days (from ED5-15). Intriguingly, these results echo similar findings from, older literature that documented that embryonic pigeons (*Colomba livia*) (Prinzinger and Hinninger, 1992) and embryonic chicks (Barnwell, 1960; Johnson, 1966) had greater rates of oxygen consumption at midday than they did at midnight. However, neither of these previous three studies detail the photoperiodic regime during egg incubation. Diurnal variation in gas exchange indicates that other metabolic processes that use and expel these gases may show rhythmicity, and indeed there is evidence in support of this. For example, the livers of ED18 chicken embryos incubated in LD display rhythmicity in the concentration of essential metabolites (triglycerides, fatty free acid and total cholesterol), whereas embryos incubated in DD did not display this rhythmicity (Wang et al., 2024). Interestingly, Wang et al. also found significant diurnal variation in the regulation of genes involved in various lipid metabolism pathways, phosphorylation, and meiotic division, indicating that the clock may use these genes as central mechanisms in regulating embryonic lipid metabolism. The driver for rhythmic changes in metabolism includes rhythmicity in clock gene expression (Marcheva et al., 2013), but feeding rhythms also play a critical role (Weger et al., 2021). Subsequently, avian embryonic lipid metabolism in the liver may be driven by both rhythmicity in clock gene expression and by local entrainment to embryonic nutrient rhythms in yolk uptake and metabolism. While there are a number of studies in adult birds that show feeding rhythms can entrain behaviour (feeding rhythms and locomotor activity) (Hau and Gwinner, 1992; Hau and Gwinner, 1996; Reierth and Stokkan, 1998) and even whole organism metabolism (body temperature and oxygen consumption) (Phillips et al., 1993), local tissue entrainment from nutrient intake is yet to displayed in either an adult or embryonic bird.

If avian embryos display rhythmicity in gas exchange, rhythmicity may also be present in the metabolic systems that facilitate gas exchange to their tissues, including cardiac function. In adult organisms, regulation and rhythmicity of cardiac parameters such as blood pressure, heart rate, and metabolism ensure proper cardiac function and resilience to changes in organismal activity levels (Dzialowski and Crossley, 2022). Avian embryos may benefit from similar rhythms when phasing developmental processes and events, but this remains largely untested. Zebrafish embryos display rhythmicity in heart rate when incubated under LD conditions (Fong et al., 2021), whilst Murray river turtle embryos (*Emydura macquarii*) incubated under DD and constant temperature also display diurnal variation in heart rate (Loudon et al., 2013). Together, these studies indicate that in other oviparous taxa, embryonic heart rate may not only be entrainable, but may be autonomous in constant conditions. Contrary to the above studies in fish and turtles, previous studies of chicken, emu (*Dromaius novaehollandiae*), muscovy duck (*Cairina moschata*), and zebra finch (*Taeniopygia guttata*) embryos have failed to find any circadian rhythm in embryonic heart rate (Andrewartha et al., 2011; Sheldon et al., 2018). In chicken embryos, although an infradian rhythm (a rhythm lasting longer than 24 h) from ED16-17 has been identified (discussed in Andrewartha et al. (2011)), circadian rhythmicity in heart rate is thought not to begin until days 1 and 2 post-hatch (Andrewartha et al., 2011). Interestingly, though no circadian rhythms have been documented in avian embryonic heart rate, a study on ED19 chick hearts found rhythmicity in the mRNA and protein expression of ion channels responsible for producing cardiac muscle contractions and kinases involved in insulin signalling and cardiac metabolism (Ko et al., 2010).

### Circadian rhythms in avian embryonic cellular metabolism and processes

Intrinsically, changes in whole-organism metabolism are tightly coupled to the outputs of cellular metabolism (Reinke and Asher, 2019). Cellular metabolism provides tissues with usable energy in the form of adenosine-triphosphate (ATP) produced by the mitochondria, the powerhouse of eukaryotic cells (Hill, 2015). ATP production for developing embryos is important for not just fuelling whole organism metabolism and physiology, but also in maintaining tissues, sustaining their growth, and producing usable energy for embryonic movement, behaviours, and sustaining activity levels. Accordingly, cellular energy production drives the embryonic developmental rate (Diaz-Cuadros et al., 2023). Hence, rhythmicity in avian embryonic mitochondrial function may have downstream effects on developmental timing and the rhythmicity of many metabolic and behavioural outputs. *In ovo*, ED18 chick embryos incubated under a 12:12LD cycle displayed daily variation in the concentration of proteins (DRP1, MFN2, PINK1) associated with mitochondrial fission (Chang et al., 2018). However, only DRP1 continued to express diurnal variation when exposed to DD for 2 days following entrainment. Furthermore, ED18 chick retinal cells display diurnal variation in ATP concentration, with chicks having greater concentrations of ATP during the dark phase than during the light phase (Huang et al., 2015), which may relate to the phasing of melatonin synthesis in retinal cells. In mammals, circadian rhythmicity in various mitochondrial dynamics have been identified (De Goede et al., 2018) and KO of clock genes in rodents can have cascading effects on mitochondrial function and dynamics (Jacobi et al., 2015; Kohsaka et al., 2014; Neufeld-Cohen et al., 2016; Peek et al., 2013; Woldt et al., 2013). However, in some cases, it appears that the effects of KO clock genes can be mixed and vary across tissues and studies (reviewed further in De Goede et al., 2018). Consequently, though some the effects are variable, there is indication that there is a tight coupling between clock gene expression and mitochondrial function. Though there is indication that diurnal variation exists in avian embryonic cellular metabolism, it is yet to be determined whether there is any circadian rhythmicity in mitochondrial function.

Beyond mitochondrial metabolism, the molecular clock interacts with various other metabolic and cellular processes. Regulation of the cell cycle (Dekens et al., 2003) and expression of genes responsible for regulating and influencing cell cycles (Laranjeiro and Whitmore, 2014; Tamai et al., 2012) display diurnal variation in zebra fish larvae and embryos (both *in vivo* and *in vitro*) indicating the importance of rhythmic light cycles for tissue maintenance and growth. Rhythmicity in adult songbird cellular proliferation has been recently identified (Hodova et al., 2025) but to our knowledge rhythmicity in cell cycles is yet to be identified in developing avian embryos. Furthermore, considering the tight link between mitochondrial function and clock genes, oxidative stress, the imbalance between free radicals and the body’s ability to neutralise them, may be tightly linked (Mezhnina et al., 2022; Wilking et al., 2013). Indeed, diurnal variation in enzymes relating to oxidative protection have been identified in many tissues of chickens such as the lung (Albarran et al., 2001), liver (Albarran et al., 2001; Maurice et al., 1991), kidney and duodenum (Maurice et al., 1991), and in various regions of the brain (Pablos et al., 1998; Albarran et al., 2001). For avian embryos, rhythmic production of free radicals from rhythmicity in mitochondrial respiration may require the coordination and timing of other metabolic pathways to regulate and neutralise free radicals which could have implications for growth and preventing damage to molecules and importantly new tissues during development.

## Potential for adaptive programming from embryonic circadian entrainment

At least in some avian species, cycles of light and dark have the potential to impact early developmental patterns, through the impacts of rhythmic gene expression, on endocrine function and both cellular and whole organism metabolism before hatching. We see three main ways in which embryonic circadian entrainment may result in adaptive changes at hatching.

a) Timing the embryonic hatching process to ensure emergence occurs at a time which maximises individual fitness.
b) Adaptively priming embryonic endocrine systems and metabolic processes with diurnal rhythms which are appropriate for the post hatch environment.
c) Adaptively priming appropriate behavioural activity and sleep patterns for the post hatch environment.

### Does circadian entrainment influence the timing of avian hatching?

As the first post-hatch day represents an extremely vulnerable developmental time window, one might predict that hatching animals should be under selection to emerge at a time which maximises their chances of survival, to avoid predation and maximise food intake. In other taxonomic groups the timing of emergence is recognised to be under selection. Indeed, the circadian rhythms in *Drosophila* eclosion have long been recognised and are thought to occur in relation to daily variation in humidity and temperature which impact on adult survival post-hatch (Pittendrigh, 1967; Pittendrigh and Skopik, 1970). The gating of emergence is routinely used as an index of the establishment of rhythmic gene expression prehatch (Saunders, 2002) and is directly controlled by the circadian clock. Interestingly, *Cry1* mutants of the silk moth (*Bombyx* mori) show no hatching rhythms in comparison to wild type embryos, which show a free-running hatching rhythm (Yuan et al., 2023) emphasising the tight coupling between the clock and hatching in insects. Furthermore, artificial selection on *Drosophila* hatch timing on the basis of the peak of population emergence timing results in a population not only with greater hatch synchrony, but also alters individual motor activity as well as clock period activity (Kannan et al., 2012b) In many insect species hatching synchronisation is significantly increased when larvae develop under cycling light and darkness (Itoh and Sumi, 2000; Matsuda, 2023; Schilman et al., 2009). Cephalopods also show rhythmic hatching patterns and increased hatching synchronisation when in the presence of cycling light (Paulij et al., 1991), whilst in some fish species hatching rhythms show sensitivity to the photoperiod they are entrained to throughout development (Villamizar et al., 2013). There is also evidence for hatch timing to be optimised across species according to ecological relevance and the activity patterns at emergence. For example, diurnal zebrafish display hatching rhythms during the light phase with a diurnal acrophrase (Villamizar et al., 2013), whilst, in contrast, the nocturnal Senegalese sole (*Solea senegalensis*) displayed rhythmic hatching during the dark phase of the LD (Villamizar et al., 2013). Interestingly, this study also showed that constant conditions (LL or DD) did not abolish hatching rhythms but rather modulated them to ultradian (a rhythm less than 24 h) bouts. However, this was more pronounced in zebrafish than Senegalese sole (Villamizar et al., 2013). These data suggest that although hatching is highly synchronised to LD cycles, in constant conditions hatching still persists in these species, hinting at an uncoupled circadian oscillator. Peaks in hatching or birth rates in both insects (Saunders, 2002) and fish (Villamizar et al., 2013), and even in primates and humans (Declercq et al., 2023; McFarland et al., 2022) therefore occur at times which plausibly maximise the survival of young. In *Drosophila,* the molecular mechanisms ‘gating’ larval emergence to prevent hatching at a time when larvae are vulnerable to predation and desiccation are now well understood (Mark et al., 2021). Considering the mechanisms controlling circadian rhythms in emergence are likely to be highly conserved, it seems surprising there has been remarkably little research focus on the timing of hatching in birds and its ecological relevance.

#### What do we know about hatch timing in wild birds?

To date, the diurnal timing of avian hatching has received remarkably little attention (Skutch, 1952; Whitman, 1919), although it seems likely that avian embryos are also under selection to hatch at the best time to maximise survival. For precocial species, synchronisation of hatching is vital to allow an entire clutch to move safely together away from the nest site (Clark and Wilson, 1981; Lack, 1968). Altricial species do not move at hatch, but embryos may be under similar selection pressures to time their hatching appropriately to allow offspring to receive appropriate levels of parental investment within the nest environment. Altricial and semi-altricial species also show higher levels of hatching asynchrony (Magrath, 1990). Early observations of hatch times in a variety of wild and domesticated (altricial) dove species (Columbidae) by Whitman (Whitman, 1919) suggest that hatching is restricted to the “forenoon”. However this observation was not formally analysed. Similarly, Skutch (1952) observed that a number of wild finch, tanager, warbler, and flycatcher species in Costa Rica (9° latitude) tend to hatch in the morning. Although these observations (N = 93 eggs, 14 species) were not sufficient for statistical analyses, patterns of hatching distributions were non-random and in many species eggs tended to hatch before midday (Skutch, 1952). Concurring with this pattern, Schrantz reported that 110/119 eggs of eastern yellow warblers (*Dendroica aestiva*) laid at Iowa Lakeside Laboratory (43°N) hatched in the early morning or night time (Schrantz, 1943). However, these reports may neglect documentation of hatching during the night, in part because documenting hatching at night has been challenging until the advent of infrared lighting (Clark and Wilson, 1981). A recent study of wild great tits (*Parus major*) in Wielkopolski National Park, Poland (52°N) showed a preference for hatching in the morning as 44% of eggs had hatched 6 h after sunrise, with the earliest hatching approximately 5 h before sunrise (Surmacki and Podkowa, 2022). Importantly, this study also documented that chicks that hatched earlier in the day were heavier on day 2 than those that hatched later in the day (Surmacki and Podkowa, 2022), suggesting that there may be selection pressures acting on hatching time, even in this altricial species. Together, these studies suggest that in passerine species hatch timing may not be random, due to selection on the ability to emerge at the time of day which maximises post-hatch survival.

Avian hatching is multi-phased, commencing hours, if not days, before external pipping is apparent (Oppenheim, 1972). Hatching involves a series of stereotyped developmental phases involving the embryo positioning within the egg, tucking its head under its right wing, and raising its beak to the air cell (Deeming, 2002). Internal pipping (breaching the air sac) occurs approximately 2 days in chickens before hatching, whilst external pipping (initial breaking of the external shell) occurs about 24 h before final emergence from the shell. The hatching process is a metabolically demanding process in which avian embryos experience multiple bouts of increased metabolic rate at internal pipping and as the chick hatches (Visschedijk, 1968). Additionally, hatching requires the development of specialised neck musculature to allow bracing, as embryos enlarge the initial eggshell opening and push down with the feet and neck to emerge (Fisher, 1958; Fisher, 1961). Given the sequence of events required to occur before emergence, if emergence time is to be optimised, then selection may act on the initiation of the phases leading to emergence. It seems highly possible that circadian rhythms in environmental factors, such as light and temperature, drive the development of mechanisms such as rhythmic gene expression during avian ontogeny. These responses may then influence the start of processes that determine the final hatch timing in avian embryos. More research is needed to determine the patterns of diurnal hatch timing in both precocial and altricial species and whether environmental cues lead to adaptive embryonic entrainment for optimising emergence timing.

### Does circadian entrainment adaptively prime embryonic endocrine and metabolic processes for the post hatch environment?

Endogenous adult clocks exist because of their adaptive significance, which confers benefits in terms of the diurnal changes in physiological parameters which adaptively influence behaviour, reproduction and fitness (Krittika and Yadav, 2020). Therefore, the adaptive significance of the priming of any embryonic clock would lie in the advantages to the emerging nestling bird in being able to time its physiological processes appropriately. The ontogeny of circadian rhythms in endocrine (e.g., GC, thyroid hormones, melatonin) and metabolic (e.g., production of antioxidants and energy) processes would be determined by the integrated benefit to the hatchlings. Indeed, two aspects of clock development would be important for the adaptive programming of young birds: synchronisation of the optimal clock timing and the amplitude of the phases, both of which will gradually develop during the ontogeny of the physiological systems (Zeman and Herichová, 2011). For example, synchronisation of biological, endocrine and physiological rhythms may ensure the appropriate timing of processes to match the post hatch environment for primed and optimal function, whilst a greater amplitude in the physiological and metabolic outputs at hatch produced from greater phase synchronicity of cells result in more coordination and efficiency in the rhythmic output. To the best of our knowledge whether this occurs remains largely untested in birds.

The main research on the adaptive significance of embryonic circadian rhythms has been conducted on insects, where the close association between the circadian clock and adult life history traits, such as development time and lifespan have not only been demonstrated, but the genes under selection have to some extent been identified (Krittika and Yadav, 2020). Likewise for birds, emergence into a world with a physiological system primed for appropriate cycles of digestion, sleep, motor activity and limited thermoregulation, still seems likely to be under selection. Yet collectively, disruption to the endocrine rhythms (e.g., melatonin and GC secretion) may result in avian embryos having less regulated and coordinated circadian and biological processes, which may have developmental consequences. For example, circadian rhythms in avian GC levels (Huffeldt et al., 2020; Rich and Romero, 2001; Schwabl et al., 2016) serve to mediate optimal metabolism for anticipated adult activity patterns, but exposure to arrhythmic light patterns, as a result of light pollution, for example, may affect the circadian rhythms in adult birds (Grunst and Grunst, 2023; Helm et al., 2024).

### Does circadian entrainment adaptively prime embryonic behavioural activity and sleep patterns?

Adaptive programming of nestlings by circadian rhythms would allow embryos to hatch with optimised levels of motor activity and rest for the diurnal schedule into which they emerge. Day old chicks need to seek food at times when it is available and avoid unnecessary energy expenditure. We hypothesise that prenatal establishment of the integrated neuroendocrine pathways underlying activity and metabolism which enable nestling birds to actively solicit food from their parents (altricial species) or to move to find food and avoid predators (precocial species) potentially with their siblings, will maximise survival in a vulnerable developmental window. Indeed, it is well established that chickens are able to walk within hours of hatching and show locomotor activity in the final third of incubation, which resembles walking although the diurnal patterns in this behaviour remain unquantified (Ryu and Bradley, 2009).

Fundamental tests of the relevance of light dark cycles during incubation for adaptively programming activity and rest require manipulations of incubation light regimes and the quantification of the impact on activity and sleep at hatching. For example, zebrafish larvae incubated under rhythmic light patterns experienced greater amplitude of behavioural rhythmicity during the light phase than the dark whereas larvae held in DD conditions experienced dampened activity rhythms (Di Rosa et al., 2015). These data suggest that light during embryonic development may play a critical role in programming the partitioning of activity rhythms in early life. yet how activity rhythms may differ in young birds after hatch is untested. Intriguingly, it would also be interesting to look across species, to compare diurnal and nocturnal species, however, to our knowledge, there are no existing studies testing circadian rhythms in nocturnal avian embryos as the few studies that do test nocturnal avian circadian rhythms have focussed on adult physiology and locomotor activity (Murakami et al., 2001; Van’t Hof et al., 1998; Wikelski et al., 2006). Current literature suggests that nocturnal birds lack strong melatonin rhythms, to enable flexibility in their activity patterns (Wikelski et al., 2006). This suggests that the adaptive benefit of development of endogenous rhythms in nocturnal birds may differ from diurnal species.

## Discussion and future directions

**FIGURE 3:**
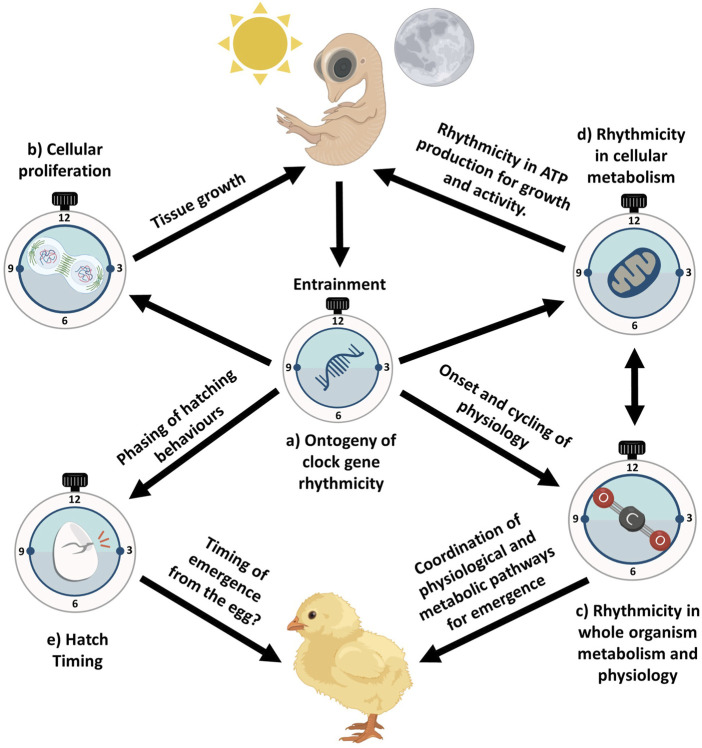
Central to all biological rhythms are clock genes which rhythmically express and dynamically shape the rhythmicity of a range of biological outputs. The ontogeny of rhythmic clock gene expression **(a)** throughout avian embryonic development may adaptively benefit embryos by stimulating rhythmicity in biological outputs influencing embryonic development and developmental events. Development of rhythmic clock gene expression may allow avian embryos to phase whole organism metabolism **(c)** which inherently shapes metabolism at the cellular level **(d)**, influencing rhythms important for phasing growth, yolk acquisition, and tissue-specific processes. Concurrently, rhythmicity in clock gene expression may shape cellular proliferation **(b)**, not shaping the rate of embryonic development but rather appropriately phasing rhythmicity in tissue growth. This rhythmicity in tissue growth would be sustained through rhythmicity in ATP generation **(d)**. Circadian entrainment by the embryo may also allow birds to time the various behavioural stages of hatching **(e)**. This coupled with coordination in changes of physiological and metabolic pathways **(c)** important to hatching may allow avian embryos to time emergence from the egg. Subsequently, embryonic entrainment and the ensuing ontogeny of rhythmicity in clock gene expression may adaptively benefit avian embryos in timing important developmental processes and events. Created in BioRender and using images from Ooid. Wellard, C. (2025) https://BioRender.com/6r4jx4a.

In this review, we have summarised the existing literature on the ontogeny of clock gene expression across taxonomically diverse embryonic systems to infer the likely development of the avian molecular clock and how this may adaptively benefit embryos, through the phasing of biological processes and developmental events (Figure 3). By assessing the mechanisms that may influence clock gene expression ontogeny, we will have a better understanding of how early exposure to rhythmic light may shape avian embryonic development. Integrating a mechanistic understanding of avian chronobiology in the context of avian ontogeny will allow an understanding of the scope of early developmental plasticity to optimise the early phenotype of birds.

### Integrating ontogeny of circadian rhythms and avian ecology

To understand the potential for rhythmic light cycles to influence early avian development, a broader range of comparative studies using carefully controlled experimental manipulations is required. For example, experimental manipulation of incubation lighting regimes has been shown to impact the pre and post hatch growth and melatonin production of precocial chicks (Özkan et al., 2012a; Özkan et al., 2012b). In addition, more ecologically relevant studies are needed of species which are exposed to a range of lighting conditions during incubation (e.g., cavity nesters, open nesters), to quantify ontogeny of clock gene expression in avian embryos (Figure 3a). Inter-specific variation in incubation and nesting strategies, such as a comparison of the relevant light cues to cavity nesters compared to open nesters, may demonstrate interspecific differences in circadian entrainment sensitivity. This would be especially interesting to study in species with extremely unique nesting strategies and conditions such as Megapodiidae species. The relevance of light cues during incubation for avian development represents an exciting avenue of research. Furthermore, the relevance of non-photic *zeitgebers* during incubation has already shown promising results from previous work in temperature entrainment in avian embryos (Zeman et al., 2009). Considering how sensitive avian embryos are to shifts in their external environment, avian embryos may potentially use a variety non-photic *zeitgebers* during embryonic entrainment, such as changes in the nest-microenvironment, for example, humidity, sound or vibrational cues generated by diurnal changes in parental incubation behaviours (e.g., egg turning). A clear understanding of the interspecific variation in light, thermal environments and incubation behaviours during early development seems likely to be key for predicting the ecologically relevant *zeitgebers* during early development. It would be especially interesting testing clock gene entrainment in avian embryos when in the presence of multiple *zeitgebers* as they would experience naturally. How do avian embryos prioritise entrainment signals in the presence of multiple weak *zeitgebers*? Would there an additive impact of multiple *zeitgbers* on the ontogeny of clock genes in embryos?

If there is inter-specific variation in the circadian organisation of birds (discussed further in Cassone, 2014), then there may be inter-specific variation in the ontogeny of the internal molecular clock. Ecologically, it would be interesting to test how evolution has driven clock gene expression ontogeny according to variation in life-history traits. For example, altricial species seem likely to have a much less developed internal clock compared to precocial species at hatch (Starck and Ricklefs, 1998), but this remains to be conclusively demonstrated. It would also be interesting to investigate the development of circadian timing in relation to both developmental rate and lifespan. In addition, as circadian timing links into the annual cycle through modulating endocrine mechanisms in relation to day length (Helm et al., 2024), it would be interesting to compare the development of clock gene expression in seasonal and non-seasonal breeders, as well as migratory and non-migratory species to determine if functional differences in the adult clock are reflected in differences in early ontogeny.

### Integrating ontogeny of circadian rhythms in avian embryonic physiology and hatching

Ultimately, rhythmicity in clock gene ontogeny may shape the transition of life stages in birds (Figure 3e). Considering the circadian influence on the transition of life stages has been established for many other oviparous taxa, avian studies are needed. *Drosophila* selected for precise hatch timing show greater stability in their hatching synchrony and subsequent activity rhythms, compared to control flies in semi-natural conditions (Kannan et al., 2012a). This prompts the question about whether avian populations primed to hatch within certain temporal windows are also primed for changes to their diurnal activity rhythms? There is also evidence in some fish species that embryos appear to favour hatch gating to reflect species-specific periods of wakefulness (Villamizar et al., 2013), whether there is interspecific variation in hatch timing across avian species in relation to species periods of wakefulness or across different breeding conditions is unknown. For example, avian species in constant light environments, such as in extreme polar environments, display considerable behavioural rhythms (e.g., incubation rhythms) under constant light (Bulla et al., 2016). Would there still be any fitness benefit to timing when to hatch in constant daylight? If there is selective benefit to timed hatching relative to dawn, it seems likely that latitude may play an important role in determining the selective benefit. Hence, testing for any latitudinal variation in diurnal hatching timing may prove a fruitful avenue of research.

### Anthropogenic disruption to avian embryonic circadian rhythms

Artificial light at night (ALAN) is a pervasive pollutant that drives a myriad of effects in de-synchronising and disrupting circadian and circannual cycles in adult birds (Gaston et al., 2017; Grunst and Grunst, 2023; Helm et al., 2024; Helm and Liedvogel, 2024). Studies of noise pollution have found prenatal noise exposure can drive changes in the developmental trajectory of birds, with these impacts being both long-lasting into adulthood and having transgenerational effects (Meillère et al., 2024). Unlike noise pollution, the developmental impacts of ALAN on birds are less clear, (Dominoni et al., 2021; Grunst et al., 2019). Exposure to ALAN throughout avian embryonic development may disrupt the natural circadian entrainment of core oscillators, de-synchronising and disrupting rhythmicity in the ontogeny of clock gene expression in both core and peripheral oscillators. This would have downstream consequences for avian embryos, potentially disrupting circadian rhythms in metabolism (e.g., ATP production, cardiac and liver biochemical processes) and physiology (e.g., disrupting melatonin and GC synthesis). But given that the avian clock likely starts to tick well before hatching, disruption of circadian rhythmicity in the HPA axis as a result of light pollution is particularly likely for precocial birds, which develop the HPA axis prior to hatching (Jenkins and Porter, 2004). Studies of the impact of light pollution on corticosterone production in wild birds report mixed results (Grunst and Grunst, 2023), but this may be because of disruption of the circadian clock, which may result in changes to the diurnal patterns of GC secretion. Interestingly, a recent study of diurnal variation of corticosterone production in thick-billed murres (*Uria lomvia)* (Huffeldt et al., 2020) reports little evidence of any diurnal rhythm in corticosterone production in birds living in the constant light conditions of a polar day. These results suggest that diel patterns of glucose mobilisation are adaptively shifted according to the requirements placed by light availability and seem likely to be driven by changes in rhythmic clock gene expression.

Although a developed clock is not a prerequisite for development, there are clearly adaptive advantages to having a developed clock that could influence the developmental trajectories and subsequent fitness of embryos and newly hatched chicks. Yet, to our knowledge there are no studies testing the developmental effects of ALAN on avian embryonic clock gene expression, ontogeny, physiological rhythms, and if there are any lasting consequences in early and adult life. Intriguingly there is conflicting evidence as to whether ALAN negatively influences the development and body condition of altricial nestlings (Grunst et al., 2020; Injaian et al., 2021; Ziegler et al., 2021). Studies should attempt to disentangle these effects through careful experimental design by separating the exposed ALAN eggs from nests during incubation to mitigate confounds (e.g., parental behaviour, food availability). Furthermore, studies should attempt to quantify and compare the effects in species with varying life history traits (altricial vs. precocial species) as current studies focus on altricial species. This area of research has considerable relevance to understanding the disruption of adaptive developmental trajectories in birds.

## Concluding remarks

For almost all organisms on Earth, the rhythmic expression of clock genes has a profound impact on the biological function of their physiological and biochemical systems, as well as their behavioural outputs (Bell-Pedersen et al., 2005). Since the characterisation of avian clock genes (Yoshimura et al., 2000), a plethora of studies have identified the pathways with which these genes may act and the ecological implications on adult avian ecology (Cassone and Westneat, 2012; Helm et al., 2024; Le Clercq et al., 2023). Developing birds have remarkable sensory capabilities to detect and respond to changes in their external environment (Rivera et al., 2018). However, to date, studies have neglected to peruse how the timing, onset, amplitude, and organismal coordination of clock gene expression across tissues may ultimately influence the fitness of individual avian embryos. Specifically, we speculate that rhythmic expression of clock genes may shape biological rhythms and processes during early development, potentially shaping individual phenotypic plasticity and fitness of avian embryos. Pursuing this exciting avenue of potential research may allow ecologists to further understand how selection has acted on the complex relationships between the molecular clock, development, and fitness in wild birds. Such an understanding may help us to understand the potential impacts of anthropogenic change on avian developmental patterns.
